# Effects of physical exercise on blood pressure during pregnancy

**DOI:** 10.1186/s12889-022-14074-z

**Published:** 2022-09-12

**Authors:** Zhu Zhu, Hang Xie, Shiping Liu, Ruizhe Yang, Juan Yu, Yiping Yan, Xu Wang, Zhihua Zhang, Wu Yan

**Affiliations:** 1 Jiangsu Vocational Institute of Commerce, Nanjing, 211168 China; 2 Office of Clinical Research Center, Children’s Hospital of Nanjing Medical University, Nanjing, 210008 China; 3 Department of Orthopedics, Children’s Hospital of Nanjing Medical University, Nanjing, 210008 China; 4 Department of Public Health, Children’s Hospital of Nanjing Medical University, Nanjing, 210008 China; 5 Department of Medicine, Gansu University of Chinese Medicine, Dingxi, 743000 China; 6 College of Mathematics and Statistics, Northwest Normal University, Lanzhou, 730070 China; 7 Department of Endocrinology, Children’s Hospital of Nanjing Medical University, Nanjing, 210008 China; 8 Yunyang People’s Hospital of Danyang, Danyang, 212300 China; 9 Department of Children Health Care, Children’s Hospital of Nanjing Medical University, Nanjing, 210008 China

**Keywords:** Pregnancy, Exercise, Blood pressure, Randomized controlled trials, Meta-analysis

## Abstract

**Objective:**

Effect of physical exercise on pregnant women currently has become a hot topic in prenatal health care. In this study, A meta-analysis was conducted on account of Randomized Controlled Trial (RCT). It focused on evaluating the effect of physical exercise intervention on blood pressure so that could provide certain evidence for health care during pregnancy.

**Methods:**

Results of relevant studies were retrieved from PubMed, Embase, Web of Science and the Cochrane Library, and all of these included studies were evaluated according to the Cochrane collaboration’s tool for assessing the risk of bias. Stata 15.1 was used for meta-analysis, and mean difference (MD) was used as statistic for pooled analysis. The effect values were combined by conventional meta-analysis and Bayesian meta-analysis respectively, and the consistency of pooled results was considered as well.

**Results:**

A total of 18 RCT studies were included in the quantitative analysis. The conventional meta-analysis showed differences in blood pressure between intervention group and control group (*P* < 0.05). Systolic and diastolic blood pressures of intervention group were 3.19 mmHg (95% *CI*: -5.13, -1.25) and 2.14 mmHg (95% *CI*: -4.26, -0.03) lower than that of control group, respectively. Bayesian meta-analysis showed that both systolic and diastolic pressure among intervention group decreased by 3.34 mmHg (95% *CrI*: -5.15, -1.56) and 2.14 mmHg (95% *CrI*: -3.79, − 0.50), respectively. Subgroup analysis supported that as long as healthy pregnant women participated in exercises, their blood pressure could be slightly regulated, while hypertension susceptible pregnant women significantly lowered blood pressure.

**Conclusion:**

Exercise intervention during pregnancy is beneficial to lower or normalize blood pressure, and this research provides clues for follow-up studies.

## Background

Physical exercise is a planned, repetitive, purposeful and systematic activity that aims to improve or maintain physical fitness [1]. Physical exercise helps reduce the risk of obesity, diabetes, high blood pressure and mental health problems in adults [2, 3]. The direct and indirect costs of physical inactivity are estimated about €910 million among 10 million people per year globally [3]. Physical inactivity is the fourth largest risk factor for death globally [4]. The American Heart Association (AHA) highlights sedentary behavior and inactivity as major risk factors for cardiovascular disease [2]. A large number of research also confirmed the value of exercise in prevention and treatment of cardiovascular disease. Physical exercise has the positive role on the primary and secondary prevention of coronary heart disease (CHD), and sedentary behavior might lead to metabolic as well as cardiovascular disease [5, 6]. A meta-analysis of 44,370 subjects found that 30 to 40 minutes of moderate physical exercise a day could offset the adverse effects of sitting for 10 hours and reduced all-cause mortality [7]. A meta-analysis based on individual participant data from randomized trials showed that less weight gain occurred in the intervention group than control group (mean difference − 0.70 kg, 95%CI: − 0.92 to − 0.48 kg) [8].

In order to meet the needs of maternal metabolism and fetal development, the state of the pregnant woman’s body will change [9]. The health effect of physical exercise on pregnant women has become a hot topic of pregnancy health care, however, the view is still controversial. Some studies supported that healthy pregnant women should be encouraged to carry out regular physical exercise [6, 10–12]. The American College of Obstetricians and Gynecologists encourage pregnant women to get at least 30 minutes of moderate physical exercise a day [13]. Conversely, there is also a view that pregnant women should exercise cautiously, especially at moderate or vigorous levels [14]. Excessive physical exercise during pregnancy can lead to chronic fatigue, hypoglycemia, and an increased risk of injury (i.e., low back pain or musculoskeleal injury). Significant changes in posture and shifts in centre of gravity alter maternal balance and coordination. In the past, Physical exercise is not recommended for women with high blood pressure during pregnancy because of concerns about the safety of the fetus and the pregnant woman, however, recent studies have showed that proper exercise is associated with a significantly reduced risk of gestational hypertensive disorders overall, either structured exercise or yoga have a beneficial effect for preventing the onset of pregnancy-induced hypertension (HDP) [9, 15].

Studies on the relevance between physical exercise and blood pressure during pregnancy are increasing, and the types of study design are diversified gradually [11, 16–19]. However, none of unanimous conclusions have been drawn. Randomized Controlled Trial (RCT) is generally regarded as the research design with the highest level of evidence. Therefore, a meta-analysis based on RCT studies was conducted to analyse the correlation between physical exercise interventions and blood pressure among pregnancy, with the aim of providing certain evidence for health care during pregnancy

## Methods

### Search strategy

All the data which was connected with the relationship between physical exercise and blood pressure among pregnancy had been searched from PubMed, Embase, Web of Science and The Cochrane Library since these databases constructed to July 15, 2022 by using a combination of subject and free words. In order to avoid omission, the references which were involved in the study had been further traced. The retrieval strategies are showed as follows (Table 1).

**Table 1:** Search strategies

Search	Search Terms
#1	maternal OR antepartum OR prenatal OR pregnancy OR pregnant
#2	aerobic OR sport OR exercise OR fitness OR “physical exercise” OR “physical activity” OR “motor activity” OR “strength training” OR “strengthening program” OR “exercise training” OR “muscle strength” OR “strength exercise”
#3	“blood pressure” OR “BP” OR “systolic pressure” OR “diastolic pressure” OR “SBP” OR “DBP”
#4	#1 AND #2 AND #3

### Inclusion and exclusion criteria

The inclusion and exclusion criteria were determined according to the Principles of the Cochrane Systematic Review Manual (PICOS), and the literatures were strictly screened.

Inclusion criteria:

1. The research objects are pregnant women (Population);
2. Clearly planned and organized interventions among pregnancy (Interventions);
3. After certain period of exercise intervention, blood pressure of target population has been measured and reported for comparison (Comparison);
4. Values of blood pressure in target population after exercise intervention and the standard deviations, or the values of blood pressure in target population before and after interventions and the standard deviations were reported (Outcomes);
5. The study design was a randomized controlled trial (Study).

Exclusion criteria:

1. Non-pregnant women from different populations, or pregnant women with HDP (Population);
2. Short-term studies or acute effects after exercise interventions (Outcome);
3. Review, conference abstracts, case reports and animal experiments (Study types);
4. Different studies conducted in the same population (Irrelevant).

### Data extraction

Two researchers independently screened literatures, extracted data, and then unified results. Firstly, the literatures that exported from different databases were imported into Endnote X7 (Thomson Reuters, New York, NY, USA), for automatically detecting duplicates and removing duplicates in batches.

After two rounds of screening, we conducted to determine the research which finally match the requirements: To begin with, the title, abstract and literature types of articles were browsed, and the other studies which were irrelevant to the topic were excluded. Secondly, remaining literatures were screened through the full text so that the final studies could be determined for inclusion and analysis.

### Bias risk assessment of involved studies

All involved studies were evaluated according to the risk assessment tool for bias in RCT, including seven items as described in the Cochrane Handbook for Systematic Reviews. The risk of bias was assessed by using Review Manager 5.3 (Copenhagen: The Nordic Cochrane Centre, The Cochrane Collaboration) software.

### Statistical analysis

All statistical analyses were performed in Stata/SE 15.1 (Stata Corp, College Station, TX, USA). Since the outcome variables concerned were continuous measured data, mean difference (MD) was used as the effect analysis statistics. Data from the involved studies were collated firstly in order to ensure comparability between studies.

Effect values were analysed by combining classical meta-analysis and Bayesian meta-analysis, respectively. In classic meta-analysis, statistics *Q* and *I*^*2*^ are assessed heterogeneity if the *Q* statistic corresponds to *P* > 0.1 or *I*^*2*^ < 50%, and the heterogeneity of the included studies was considered acceptable in this case, and the effect values were combined using the Inverse Variance method of the fixed effect model. On the contrary, once it comes to significant heterogeneity among studies, the effect values would be combined by random effect model. Egger’s and Begg’s tests were used to estimate publication bias and provide a visual display of funnel plots. The sensitivity analysis has been applied to exclude each study individually and evaluate it for determining the extent to any single study that contributed to overall combined effect.

Compared with classical frequency-school meta-analysis, Bayesian meta-analysis is more advantageous in dealing with complex problems such as random effects, hierarchical structure and data sparseness [20]. Especially when the sample size of the included study is small and the data does not meet normal distribution or cannot be tested for normality. Combined estimates of effects using classical meta-analysis may be biased. However, Bayesian analysis can directly calculate the accurate finite sample distribution without relying on the asymptotic theory, and fully consider the uncertainty of the model, so it can be considered that the Bayesian estimation of meta-analysis is more reliable and reasonable [21]. After improving the model estimation accuracy, the modeling method is more flexible. We used Markov Chain Monte Carlo (MCMC) method to obtain the mean value, and the variation degree between studies (*θτ*^*2*^*),* whose main command is “BayesMH”, which uses the adjusted Metropolis Hastings (MH) and Inverse gamma methods to compute the Bayesian regression model*.*

## Results

**Fig. 1:**
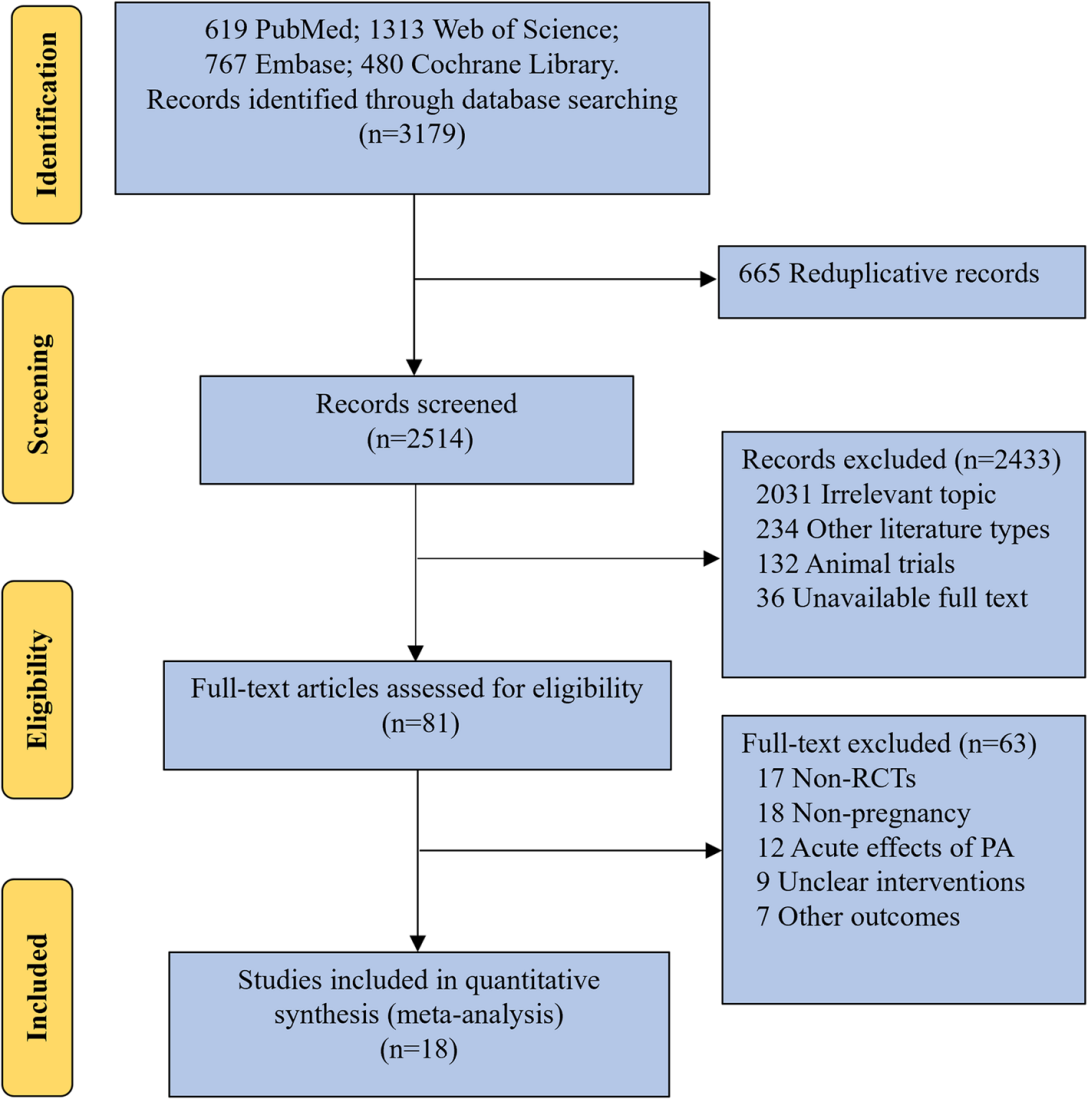
Flow chart of literature screening

**Fig. 2:**
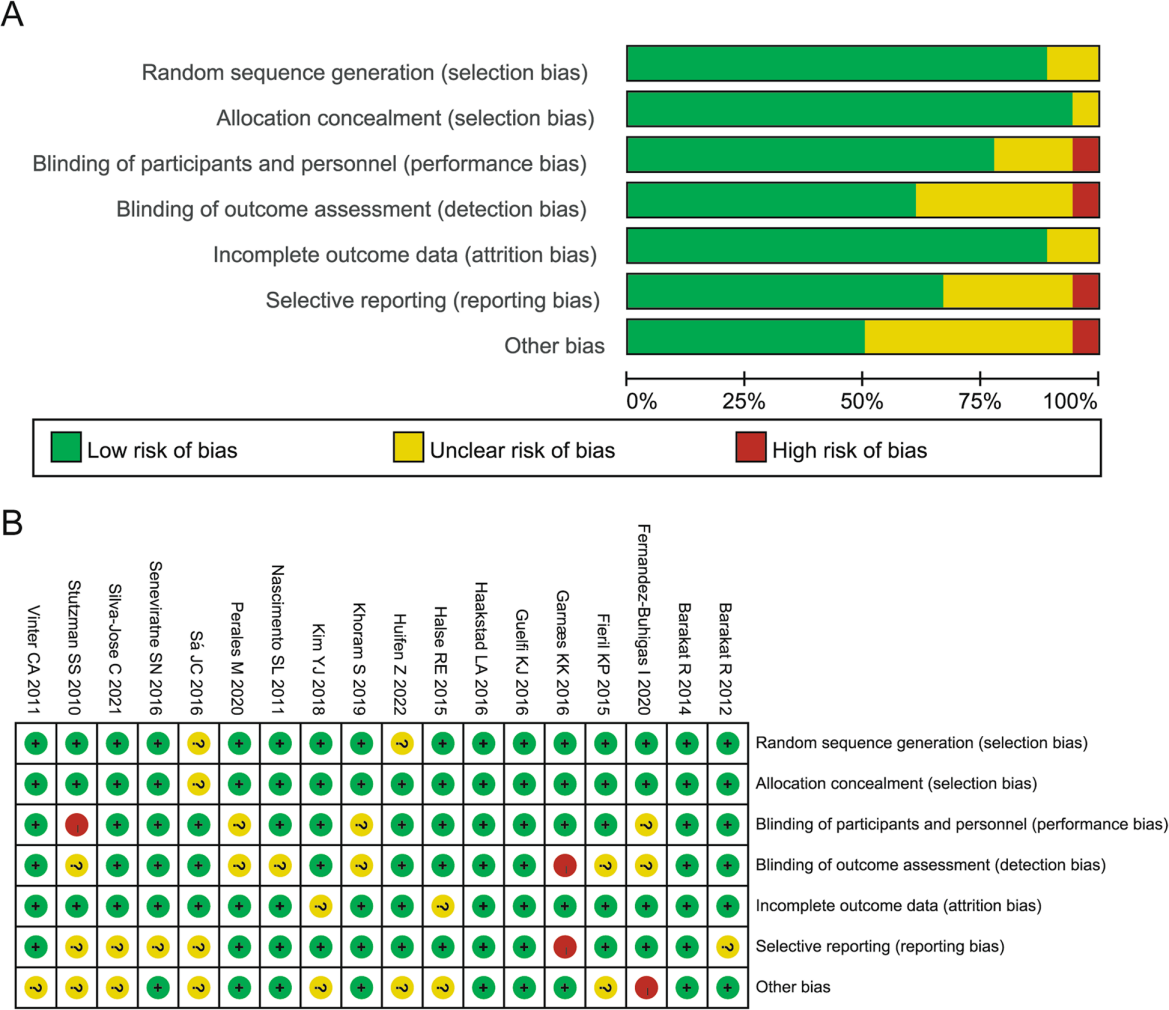
Risk of bias for studies included in the meta-analysis. **A** Assessment of the bias risk items included in the study; **B** Include the assessment of each bias risk item for each study

A total of 3179 records were retrieved from PubMed, Embase, Web of Science, and The Cochrane Library. The remaining 81 records were screened based on the title and abstract, and then the full text was read according to the inclusion criteria. Finally, 18 RCT studies that met the criteria were included in the analysis [10, 16, 18, 19, 22–35] (Fig. 1). Seven studies focused on exercise interventions in healthy pregnant women, 10 studies looked at women at high risk for HDP, including overweight or obese women, women with chronic hypertension and family history of hypertension. There is only one study focused on both groups of women (Table 2). The purpose of quality assessment review is to ensure the quality of included studies meets the requirements and to assess the risk of bias in the included studies according to the tools which are provided in the Cochrane Manual. Most studies have reported allocation hiding for sequence generation, including automatic computer generation of random sequences, the use of random number tables, and coin flipping. Although only a few included studies blinded subjects, the outcome variables of concern were not affected by the absence of blinding. Dropouts were reported in all included studies, and no selective reporting bias was found, nor other biases were shown. Therefore, the literature that included in this meta-analysis maintained a high quality (Fig. 2).

**Table 2:** Basic features of the included literature

Study (Year)	Country	Subjects (T/C)	Baseline BMI (kg/m^2^)	Age (years)	Characteristics	Interventions	Frequency	Duration
Barakat R (2012) [29]	Spain	40/43	T: 22.70 ± 2.80 C: 23.00 ± 2.90	T: 32.00 ± 4.00 C: 31.00 ± 3.00	Healthy pregnant women	Land/aquatic activities	35–45 min session, 3 times a week	Weeks (6–9) to (38–39)
Barakat R (2014) [23]	Spain	107/93	T: 23.78 ± 4.40 C: 24.09 ± 4.32	T: 31.57 ± 3.87 C: 31.51 ± 3.92	Healthy pregnant women	Physical conditioning program	55–60 min, 3 days per week	Weeks (9–13) to (39–40)
Fernandez-Buhigas I (2020) [24]	Spain	41/51	T: 22.81 ± 3.54 C: 23.80 ± 5.09	T: 33.17 ± 3.19 C: 32.63 ± 4.66	General pregnancy	Supervised physical conditioning program	30 mins per day at least 3 times per week	Weeks (12^+ 3^–15^+ 6^ to (38^+ 0^–39^+ 6^)
Fieril KP (2015) [25]	Sweden	38/34	T: 22.60 ± 2.50 C: 23.00 ± 2.60	T: 30.80 ± 3.60 C: 30.60 ± 3.40	General pregnancy	High-repetition, resistance training	2 times a week	Weeks 14 to 25
Garnæs KK (2016) [26]	Norway	38/36	T: 33.90 ± 3.80 C: 35.10 ± 4.60	T: 31.30 ± 3.80 C: 31.40 ± 4.70	Obese pregnant women	Hospital/home exercise； muscle exercises	3 times weekly; 50 mins at least once	Weeks (12–18) to delivery
Guelfi KJ (2016) [30]	Australia	84/85	<25 (85) 25 ~ 29.90 (57) ≥30 (38)	T: 33.60 ± 4.10 C: 33.80 ± 3.90	Women with history of GDM	Supervised home-based exercise program	20–30 min to a maximum of 60 mins, 3 times a week	Started weeks (12–14), lasted for 14 weeks
Haakstad LA (2016) [19]	Norway	35/26	T: 22.90 ± 3.20 C: 23.00 ± 3.10	T: 31.50 ± 3.10 C: 29.40 ± 3.80	Healthy pregnant women	Aerobic exercise	60 mins 2/week	Started weeks (12–24)
Halse RE (2015) [31]	Australia	20/20	T: 25.20 ± 6.70 C: 26.40 ± 7.10	T: 34.00 ± 5.00 C: 32.00 ± 3.00	Pregnancy with GDM	Supervised home-based exercise program	4 supervised exercise sessions each week, 2 unsupervised exercise sessions	Weeks (28.8 ± 0.9) to (34.6 ± 0.3)
Khoram S (2019) [16]	Iran	36/36	T: 27.36 ± 3.64 C: 34.97 ± 4.37	T: 31.91 ± 4.62 C: 31.00 ± 5.29	Pregnant women susceptible to GH	Walking program	20–30 min，4 times a week	Weeks 14 to 34
Kim YJ (2018) [27]	Korea	23/22	Not mentioned	T: 32.22 ± 2.58 C: 31.50 ± 4.48	High risk pregnant women	Structured bed exercise	4 times in total from the 3th day hospitalization once per day for 4 days, for 30 mins per time	Not mentioned
Nascimento SL (2011) [28]	Brazil	39/41	T: 29.70 ± 6.80 C: 30.90 ± 5.90	T: 34.80 ± 6.60 C: 36.40 ± 6.90	Overweight/ obese pregnant women	Supervised exercise; home exercise	40 mins weekly	Lasted for mean of 12.3 weeks
Perales M (2020) [10]	Spain	688/660	T: 23.50 ± 3.90 C: 23.60 ± 4.00	T: 32.00 ± 4.00 C: 31.00 ± 4.00	General pregnancy	Moderate intensity exercise program	50 to 55 mins sessions	Weeks 9 to 38/39
Sá JC (2016) [18]	Brazil	15/15	T: 2.10 ± 4.00 C: 32.30 ± 5.80	25.80 ± 4.80	Overweight/ obese sedentary PCOS women	Walking / jogging	3 times per week	Lasted for 16 weeks
Seneviratne SN (2016) [32]	New Zealand	37/37	≥25.00	18.00–40.00	Overweight and obese pregnant women	Moderate intensity stationary cycling programme	Between 15 and 30 mins per session, 3-5sessions per week	Lasted for 16 weeks
Stutzman SS (2010) [22]	Canada	11/11	T: 26.20 ± 5.99 C: 26.76 ± 5.98	T: 26.76 ± 5.98 C: 26.02 ± 4.40	Normal and overweight pregnant women	Low-intensity walking program	5 days per week	Lasted for or 16 weeks
Huifen Z (2022) [34]	China	46/43	T: 23.03 ± 5.22 C: 21.98 ± 2.96	T: 31.84 ± 5.19 C:31.35 ± 4.72	GDM women	resistance exercise of upper and lower limb muscles	3 times per week	at least 6 weeks
Silva-Jose C (2021) [35]	Spain	31/41	T:22.61 ± 3.22 C:23.06 ± 7.80	T:32.29 ± 6.36 C:33.93 ± 4.59	singleton and uncomplicated pregnancy	virtual supervised exercise program	3 weekly sessions of 55–60 min	8–10 and 38– 39 weeks of pregnancy
Vinter CA (2011) [33]	Denmark	150/154	T: 33.89 ± 3.59 C: 34.00 ± 3.89	T: 29.35 ± 3.74 C: 28.65 ± 3.74	Obese pregnant women	Not mentioned	Moderately physically active 30-60 mins daily	Lasted for 6 months

*T* intervention group, *C* control group, *BMI* Body mass index, *GH* Gestational hypertension, *GDM* Gestational diabetes mellitus, *PCOS* Polycystic ovarian syndrome

### Classic meta-analysis

**Fig. 3:**
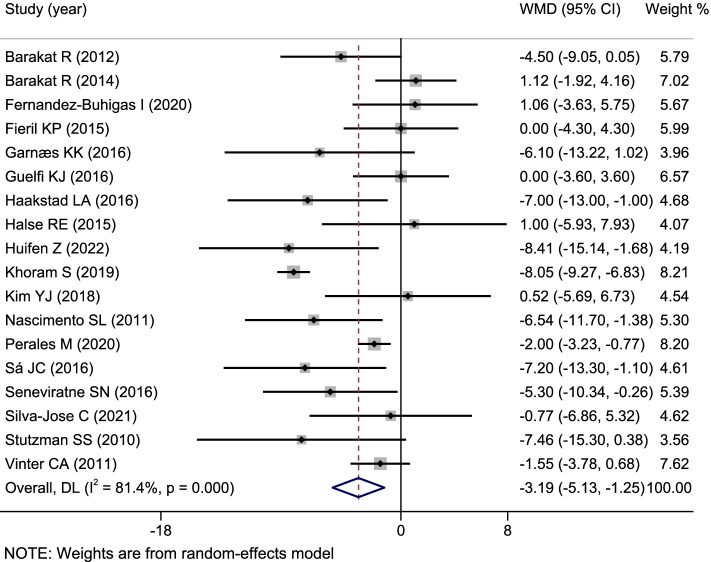
A classic meta-analysis of systolic blood pressure between the exercise intervention group and the control group during pregnancy

**Fig. 4:**
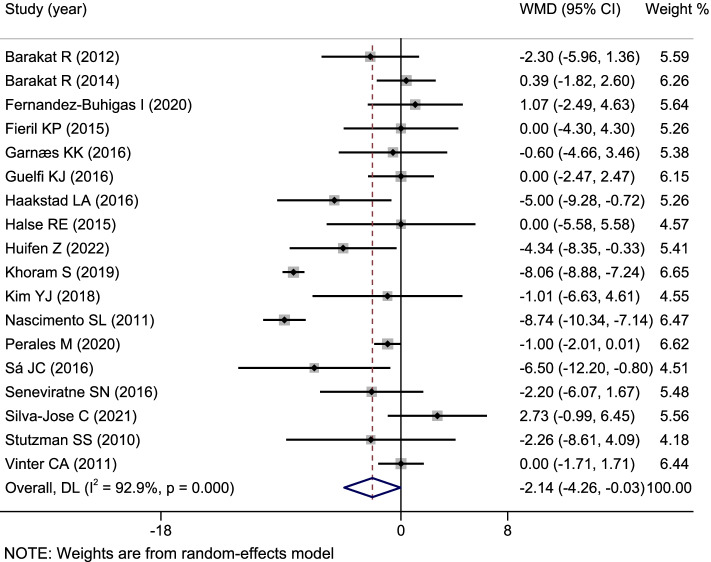
A classic meta-analysis of diastolic blood pressure between the exercise intervention group and the control group during pregnancy

A total of 18 RCTs reported an association between exercise interventions during pregnancy and changes of blood pressure, including 2930 pregnant women, which 1452 are the exercise intervention group and 1478 are in the conventional care control group. The mean and standard deviation of blood pressure of each study intervention and control group were inputted into Stata software for effect value combination. Due to the high heterogeneity among studies (*I*^*2*^ = 81.4%), we accepted the random effect model to combine the effect values. The combined results showed that, comparing with the control group, the systolic pressure of pregnant women in exercise intervention group lowered 3.19 mmHg (95%*CI*: − 5.13, − 1.25), and the diastolic blood pressure lowered 2.14 mmHg (95%*CI*: − 4.26, − 0.03), with statistically significant differences (Figs. 3 and 4)

.

### Bayesian meta-analysis

According to random MCMC method, the posterior mean difference and variance (*δ*^*2*^) and corresponding 95% *CrI*. In terms of systolic pressure, the mean difference between exercise intervention group and control group was − 3.34 mmHg (95% *CrI*: − 5.15, − 1.56). In terms of diastolic pressure, the mean difference between exercise intervention group and control group was-2.14 mmHg (95% *CrI*: − 3.19, − 0.50), which was similar to the results of above classic meta-analysis and also supported that combined effect values were stable and reliable (Table 3).

**Table 3:** Comparison of blood pressure between exercise intervention group and control group in pregnancy by Bayesian meta-analysis (mmHg)

Study	Systolic blood pressure				Diastolic blood pressure			
	Mean	Std. Dev.	Median	95% *CrI*	Mean	Std. Dev.	Median	95% *CrI*
Barakat R (2012) [29]	− 4.30	1.39	− 4.26	(− 6.99, − 1.53)	−2.29	1.25	−2.25	(− 4.76, 0.12)
Barakat R (2014) [23]	0.50	1.18	0.47	(− 1.81, 2.85)	0.08	1.01	0.08	(− 1.83, 2.07)
Fernandez-Buhigas I (2020) [24]	0.21	1.43	0.20	(− 2.59, 2.98)	0.56	1.28	0.53	(− 1.92, 3.12)
Fieril KP (2015) [25]	− 0.61	1.34	− 0.63	(− 3.19, 2.05)	− 0.46	1.35	− 0.47	(− 3.03, 2.22)
Garnæs KK (2016) [26]	− 5.38	1.68	− 5.32	(− 8.81, − 2.12)	− 0.86	1.33	− 0.86	(− 3.54, 1.73)
Guelfi KJ (2016) [30]	− 0.47	1.29	− 0.47	(− 2.95, 2.05)	− 0.22	1.07	− 0.24	(− 2.31, 1.81)
Haakstad LA (2016) [19]	−6.09	1.57	−6.07	(− 9.25, − 3.04)	− 4.40	1.37	− 4.39	(− 7.10, − 1.76)
Halse RE (2015) [31]	−0.13	1.73	−0.19	(− 3.4, 3.37)	− 0.50	1.52	− 0.52	(− 3.51, 2.46)
Huifen Z (2022) [34]	−7.77	0.77	−7.77	(− 9.26, − 6.25)	−3.97	1.32	−3.95	(− 6.53, − 1.4)
Khoram S (2019) [16]	− 0.38	1.62	− 0.40	(−3.48, 2.87)	−7.78	0.65	−7.79	(− 9.05, − 6.57)
Kim YJ (2018) [27]	− 5.88	1.44	−5.88	(− 8.74, − 3.08)	− 1.28	1.50	−1.31	(− 4.18, 1.70)
Nascimento SL (2011) [28]	− 2.09	0.82	−2.11	(− 3.68, − 0.5)	− 8.20	0.93	−8.22	(− 10.03, − 6.40)
Perales M (2020) [10]	− 6.28	1.60	−6.26	(− 9.44, − 3.2)	− 1.07	0.70	−1.06	(− 2.50, 0.27)
Sá JC (2016) [18]	− 4.85	1.45	− 4.82	(− 7.68, − 1.99)	−5.40	1.51	−5.39	(− 8.37, − 2.46)
Seneviratne SN (2016) [32]	−6.38	1.76	−6.38	(−9.99, − 3.02)	− 2.25	1.26	− 2.26	(− 4.66, 0.27)
Silva-Jose C (2021) [35]	−1.73	1.03	−1.73	(− 3.74, 0.21)	1.94	1.33	1.96	(−0.68, 4.44)
Stutzman SS (2010) [22]	− 7.13	1.73	− 7.09	(− 10.57, − 3.89)	− 2.21	1.63	− 2.22	(− 5.36, 0.94)
Vinter CA (2011) [33]	−1.35	1.53	−1.35	(− 4.29, 1.62)	− 0.16	0.90	−0.16	(− 1.90, 1.60)
***d***	**−3.34**	**0.90**	**−3.33**	**(− 5.15, − 1.56)**	**− 2.14**	**0.83**	**−2.14**	**(− 3.79, − 0.50)**
**σ**^**2**^	11.87	5.58	10.72	(4.68, 25.68)	10.63	4.65	9.66	(4.63, 22.65)

*CrI* Credibility interval, *D* mean difference, σ^*2*^ the variance

**Fig. 5:**
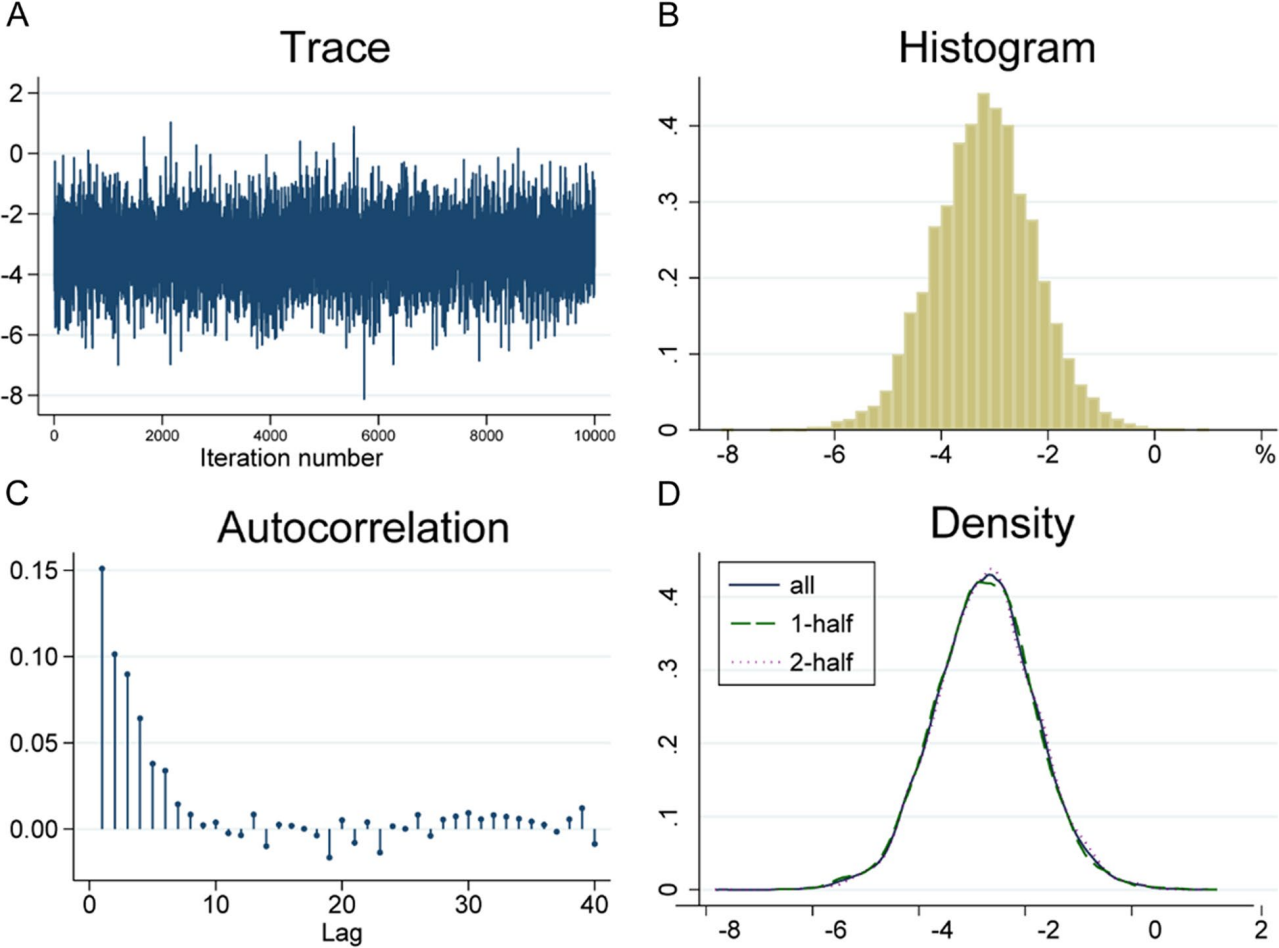
Convergence diagnosis diagram of Bayesian meta-analysis. **A** Trajectory diagram; **B** Histogram; **C** Autocorrelation graph; **D** nuclear density map

In addition, the Bayesian convergence is visualized, including trajectory graph, autocorrelation graph, histogram and kernel density graph. The trajectory diagram shows that when MCMC reaches steady state, the simulated parameter values fluctuate up and down around the mean value (Fig. 5A). The histogram is approximately normally distributed, and consistent with the marginal posterior distribution of the specified conjugate normal (Fig. 5B). The autocorrelation diagram shows a series of lag ranges for the MCMC sample autocorrelation starting with a lag of 0, at which point the value is equal to the MCMC sample variance (Fig. 5C). Kernel density curve is another way to simulate posterior distribution (Fig. 5D). Efficiency analysis indicates that Bayesian analysis has a positive efficiency for statistical inference of unknown parameters, and the prior information has been correctly verified.

### Subgroup analysis

A subgroup analysis which applied both classical and Bayesian methods indicated that exercise interventions during pregnancy can lower blood pressure slightly and moderately among ordinary healthy pregnant woman, and the Bayesian analysis did not point out any statistically significant difference. Exercise interventions can significantly reduce blood pressure in high-risk pregnant women. The results of two analysis methods are consistent, so they are considered robust and reliable (Table 4).

**Table 4:** Subgroup analysis of the relationship between exercise intervention and blood pressure in healthy pregnant women and high-risk pregnant women prone to HDP (mmHg)

	Subgroup	Studies	Conventional Meta-analysis			Bayesian Meta-analysis	
			WMD	95% *CI*	*I*^*2*^%	Difference	95% *CrI*
SBP	General	8	−1.64	(−2.64, −0.63)	30.0	−1.64	(−3.82, 0.41)
	High risk	11	−4.39	(− 7.08, −1.69)	78.2	− 4.56	(− 7.10, −2.01)
DBP	General	8	−0.68	(−1.49, 0.14)	33.6	− 0.42	(−2.08, 1.35)
	High risk	11	−3.38	(−6.12, −0.63)	91.9	− 3.36	(−5.66, −1.01)

*SBP* systolic blood pressure, *DBP* diastolic blood pressure

### Publication bias and sensitivity analysis

**Fig. 6:**
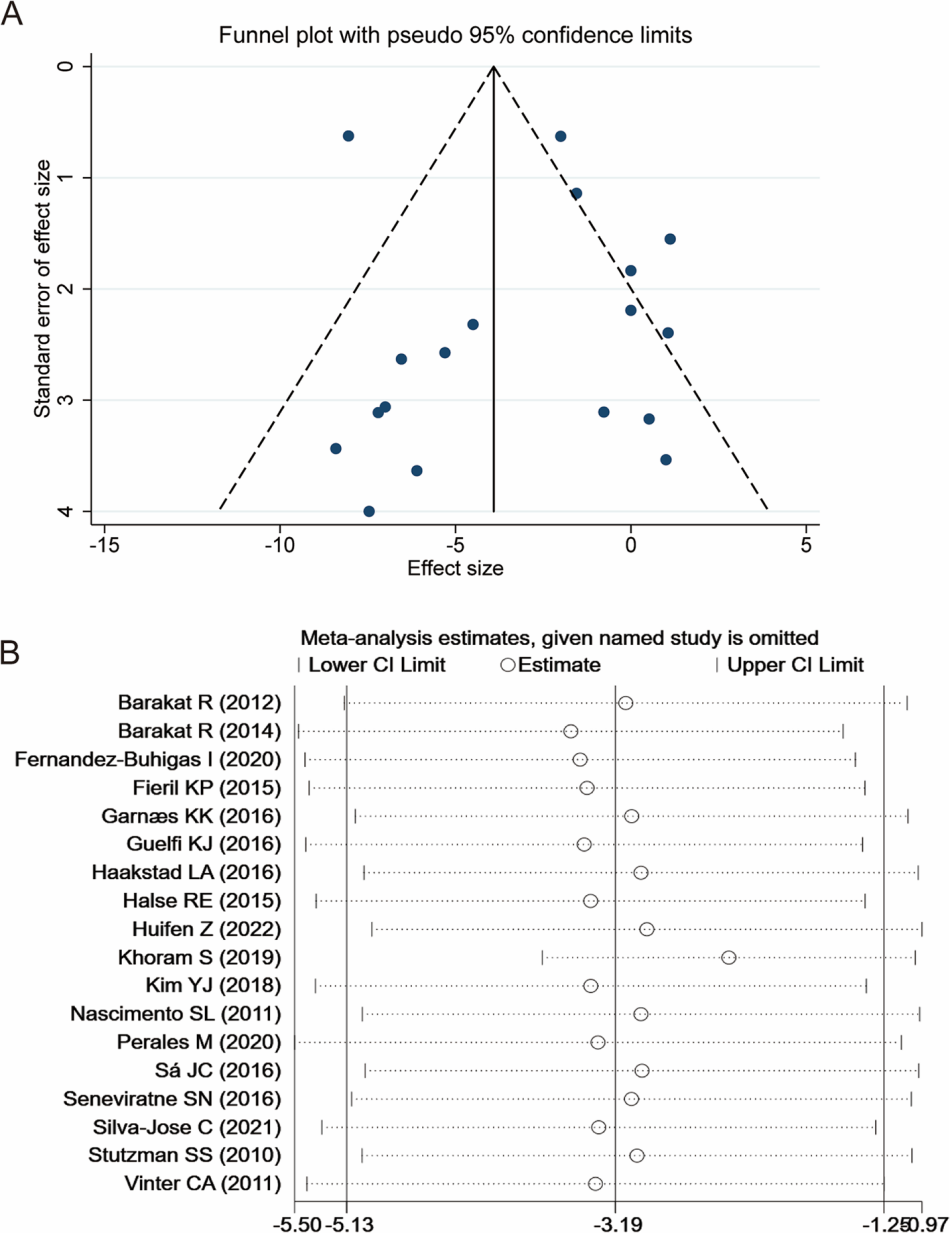
Meta-analysis publication bias and sensitivity analysis. **A** Funnel plot; **B** Sensitivity analysis

According to the publication bias test of the 18 included studies, no significant publication bias has been found on Egger’s test (*P* = 0.425), and the funnel plot was relatively symmetrical (Fig. 6A). In addition, sensitivity analysis identified that the combined effect value ES and 95%CI were basically stable before and after excluding a single study, and it provided evidence that the analysis results were reliable (Fig. 6B).

## Discussion

Regular physical exercise in normal pregnant women might be associate with health effects [36–38]. In our study, meta-analysis was conducted for the studies about physical exercise interventions and blood pressure changes during pregnancy, and the effect values were combined by the method of classical meta-analysis as well as Bayesian meta-analysis. The results identified physical exercise during pregnancy can effectively reduce blood pressure. Subgroup analysis presented that physical exercise could slightly regulate blood pressure in healthy pregnant women with normal pregnancy, and physical exercise could significantly reduce blood pressure in pregnant women with high-risk of HDP, and the effect was more obvious.

The result of this meta-analysis indicated that regular physical exercise during pregnancy is beneficial to pregnancy health. After a 12-week exercise intervention among healthy and nonactive pregnant women, the changes in resting blood pressure between intervention and baseline were assessed, and the researchers found that regular, long-term physical exercise significantly reduced resting systolic blood pressure [19]; In a recent meta-analysis, physical exercise interventions during pregnancy reduced the risk of pregnancy-induced hypertension and preeclampsia by 39 and 41%, respectively [17]. The underlying mechanism between prenatal physical exercise and blood pressure control is unclear and may be due to the effect of physical exercise on preventing excessive weight gain, reducing oxidative stress and inflammation, and improving vascular endothelial function [39, 40]. Elevated blood pressure remains a risk factor for maternal, fetus and neonatal mortality, and physical exercise during pregnancy is of particular importance for health [41].

The effect of physical exercise on blood pressure has also been shown in overweight and obese pregnant women who were susceptible to hypertension. In a study conducted in Norway, pregnant women with obesity were randomly divided into physical exercise group and control group. The pregnant women exercised from the middle to the late trimester, and the systolic blood pressure in exercise group was 7.73 mmHg lower than that in the control group at the late trimester [26]. Walking intervention has been found to reduce the incidence of HDP in high-risk pregnant women who contain family history of hypertension, preeclampsia, or chronic hypertension. Therefore, moderate physical exercise is recommended for pregnant women susceptible to hypertension in pregnancy to improve their health status [16]. In a study of hospitalized high-risk pregnant women, participants in intervention group had received lateral and supine structured bed physical exercise, and control group had maintained standard nursing. There were no significant differences in blood pressure, heart rate and fetal heart rate between two groups, but the physical discomfort and anxiety symptoms of pregnant women in structured physical exercise group had been significantly improved. Therefore, structured bed physical exercise for hospitalized high-risk bedridden pregnant women will not increase the health risks of themselves and the fetus, and bed exercise intervention can be considered in the management of high-risk pregnant women during pregnancy [27].

Daily physical exercise is recommended for non-risk or low-risk pregnant women, and regular physical exercise during pregnancy can prevent HDP [42, 43]. In studies of pregnant women with HDP, regular physical exercise during pregnancy has been associated with a reduced risk of adverse outcomes, including preeclampsia, preterm birth and cardiovascular disease [44, 45]. Mild physical exercise during pregnancy, including swimming, walking, yoga and stretching, improves blood vessel perfusion while stimulating major muscle and effectively reducing pregnancy complications [46]. Even in women who do not physical exercise before pregnancy, a moderate physical exercise during pregnancy can improve their health without affecting placental blood flow resistance and fetal growth [47].

The exercise interventions performed in studies included in this meta-analysis can be broadly divided into three types: (1) moderate walking and(or) jogging; (2) pedaling and cycling, and (3) comprehensive exercises designed according to American College of Obstetricians and Gynaecologists (ACOG), which accounts for the majority. This type of exercise program usually consists of four parts: a few minutes of warm-up; followed by aerobic exercises; resistance muscle training including upper and lower limb muscles, joint and pelvic floor muscle exercises; end with stretching and relaxation. One study included land aerobic sessions and aquatic activities session. Purpose of exercise in the water was to avoid a huge impact [29]. At present, regular participation in physical exercise is encouraged during pregnancy for healthy pregnant women [44]. Although there is a wide range of physical exercise that are currently recommended during pregnancy, there is insufficient evidence of superiority among different physical exercise [48]. It is worth noting that, the body at different stages of pregnancy will undergo significant changes, and it is necessary to timely adjust the physical exercise program [47].

This study is the first meta-analysis to explore and evaluate the relationship between physical exercise intervention during pregnancy and blood pressure by using both classical meta-analysis and Bayesian analysis. Through rigorous literature selection and quality assessment, the relevant randomized controlled trial was identified with a high level of evidence, and provided certain clues for blood pressure management during pregnancy. However, there are some certain limitations. First of all, nutritional intervention has not been considered, and its potential relationship with gestational blood pressure changes and pregnancy outcomes has not been examined. Secondly, some studies were limited to specific populations, such as GDM, overweight or obese pregnant women, and that lead to limit generality of the results.

However, we attempted to reduce heterogeneity by introducing subgroup analysis. Furthermore, low compliance in some studies may have affected the true health effects of physical exercise during pregnancy to some extent. Finally, the time of interventions in some studies was not clear, and the intervention effect in different stages of pregnancy was different, which limited the interpretation of the results.

## Conclusion

This meta-analysis presented that exercise interventions in pregnant women might reduce systolic and diastolic blood pressure during pregnancy, particularly in pregnant women at high risk for HDP. Therefore, exercise should be promoted as an important way to improve the cardiovascular health of pregnant women.

## Data Availability

The datasets generated and analyzed during the current study are available from the corresponding author on reasonable request.
